# Frequency of *CDH1, CTNNA1* and *CTNND1* Germline Variants in Families with Diffuse and Mixed Gastric Cancer

**DOI:** 10.3390/cancers15174313

**Published:** 2023-08-29

**Authors:** Joana Guerra, Carla Pinto, Pedro Pinto, Manuela Pinheiro, Catarina Santos, Ana Peixoto, Carla Escudeiro, Ana Barbosa, Miguel Porto, Inês Francisco, Paula Lopes, Ana Raquel Isidoro, Ana Luísa Cunha, Cristina Albuquerque, Isabel Claro, Carla Oliveira, João Silva, Manuel R. Teixeira

**Affiliations:** 1 Cancer Genetics Group, IPO-Porto Research Center (CI-IPOP)/RISE@CI-IPOP (Health Research Network), Portuguese Oncology Institute of Porto (IPO-Porto)/Porto Comprehensive Cancer Center, 4200-072 Porto, Portugal; joana.goncalves.guerra@ipoporto.min-saude.pt (J.G.); carla.a.pinto@ipoporto.min-saude.pt (C.P.); pedropinto18@hotmail.com (P.P.); manuelap@ipoporto.min-saude.pt (M.P.); catarinasantos@ipoporto.min-saude.pt (C.S.); analuisamoura@ipoporto.min-saude.pt (A.P.); carla.escudeiro@ipoporto.min-saude.pt (C.E.); i12756@ipoporto.min-saude.pt (A.B.); masps.porto@gmail.com (M.P.); joao.pinho.silva@ipoporto.min-saude.pt (J.S.); 2 Doctoral Programme in Biomedical Sciences, School Medicine and Biomedical Sciences, University of Porto (ICBAS-UP), 4050-313 Porto, Portugal; 3 Department of Laboratory Genetics, Portuguese Oncology Institute of Porto (IPO-Porto)/Porto Comprehensive Cancer Center, 4200-072 Porto, Portugal; 4 Department of Pathological, Cytological and Thanatological Anatomy, School of Health, Polytechnic Institute of Porto, 4200-072 Porto, Portugal; 5 Molecular Pathobiology Research Unit, Portuguese Oncology Institute of Lisbon, 1099-023 Lisbon, Portugal; mfrancisco@ipolisboa.min-saude.pt (I.F.); calbuque@ipolisboa.min-saude.pt (C.A.); 6 Department of Pathology, Portuguese Oncology Institute of Porto (IPO Porto)/Porto Comprehensive Cancer Center, 4200-072 Porto, Portugal; paulalopes@ipoporto.min-saude.pt (P.L.); anaraquel.isidoro@ulsm.min-saude.pt (A.R.I.); analuisa.cunha@ipoporto.min-saude.pt (A.L.C.); 7 Gastroenterology Department, Portuguese Oncology Institute of Lisbon, 1099-023 Lisbon, Portugal; iclaro@ipolisboa.min-saude.pt; 8 Familiar Cancer Risk Clinic, Portuguese Oncology Institute of Lisbon, 1099-023 Lisbon, Portugal; 9 i3S-Instituto de Investigação e Inovação em Saúde, 4200-135 Porto, Portugal; carlaol@i3s.up.pt; 10 IPATIMUP-Instituto de Patologia e Imunologia Molecular da Universidade do Porto, 4200-135 Porto, Portugal; 11 FMUP-Faculty of Medicine of the University of Porto, 4100-179 Porto, Portugal; 12 Medical Genetics Department, Portuguese Oncology Institute of Porto (IPO-Porto)/Porto Comprehensive Cancer Center, 4200-072 Porto, Portugal; 13 School of Medicine and Biomedical Sciences (ICBAS), University of Porto, 4050-313 Porto, Portugal

**Keywords:** hereditary diffuse gastric cancer, mixed gastric cancer, *CDH1* gene, *CTNNA1* gene, *CTNND1* gene

## Abstract

**Simple Summary:**

Hereditary diffuse gastric cancer (HDGC) is caused by germline pathogenic variants in the *CDH1* and *CTNNA1* genes and is characterized by a high prevalence of diffuse gastric cancer and lobular breast cancer. We aimed to evaluate the contribution of *CTNNA1* and *CTNND1* germline variants to HDGC, as well as to compare the frequencies of *CDH1* and *CTNNA1* (and eventually *CTNND1*) germline variants between patients with diffuse and mixed gastric carcinomas. In this study, we report a deleterious *CTNNA1* germline variant and four *CDH1* pathogenic variants in patients with criteria for genetic testing. None of the cases with mixed gastric cancer carried pathogenic variants in either the *CDH1* or the *CTNNA1* genes, so there is no evidence to use this tumor type in testing criteria.

**Abstract:**

The most well-characterized hereditary form of gastric cancer is hereditary diffuse gastric cancer (HDGC), an autosomal dominant syndrome characterized by an increased risk of diffuse gastric and lobular breast cancer. HDGC is predominantly caused by germline pathogenic variants in the *CDH1* gene, and more rarely in the *CTNNA1* gene. Furthermore, the International Gastric Cancer Linkage Consortium (IGCLC) guidelines do not clarify whether or not mixed gastric cancer (with a diffuse component) should be considered in the HDGC genetic testing criteria. We aimed to evaluate the contribution of *CTNNA1* and *CTNND1* germline variants to HDGC. Additionally, we also intended to compare the frequencies of *CDH1* and *CTNNA1* (and eventually *CTNND1*) germline variants between patients with diffuse and mixed gastric carcinomas to evaluate if genetic testing for these genes should or should not be considered in patients with the latter. We analyzed the *CDH1* gene in 67 cases affected with early-onset/familial mixed gastric carcinomas and the *CTNNA1* and *CTNND1* genes in 208 cases with diffuse or mixed gastric cancer who had tested negative for *CDH1* pathogenic germline variants. A deleterious *CTNNA1* germline variant was found in 0.7% (1/141) of diffuse gastric cancer patients meeting the 2020 IGCLC criteria, as compared to the rate of 2.8% of *CDH1* deleterious variants found by us in this setting. No deleterious variants were found in *CTNND1*, but six variants of uncertain significance were identified in this gene. We did not find any pathogenic *CDH1*, *CTNNA1* or *CTNND1* variant in index patients with early-onset/familial mixed gastric cancer, so there is no evidence that supports including this tumor type in the testing criteria for germline variants in these genes. The role of the *CTNND1* gene in inherited gastric cancer predisposition is still unclear.

## 1. Introduction

Familial clustering is observed in approximately 10% of gastric cancer (GC) cases; however, only 1–3% of GC cases result from a known hereditary syndrome [1]. The most well-characterized hereditary form of GC is hereditary diffuse gastric cancer (HDGC), which is an autosomal dominant syndrome characterized by an increased risk of diffuse gastric cancer (DGC) and lobular breast cancer (LBC) [2]. Until recently, *CDH1* was the only known susceptibility gene involved in HDGC, but, in 2013, Majewski and collaborators described a germline variant in the catenin alpha-1 gene (*CTNNA1*) for the first time in a family that fulfilled the HDGC clinical criteria [3]. Since then, to our knowledge, 13 pathogenic variants in *CTNNA1* have been described in 15 families fulfilling the HDGC criteria [3,4,5,6,7,8,9,10,11]. Additionally, loss-of-function variants have been described in families without HDGC clinical criteria [9,11]. Benusiglio and collaborators reported two asymptomatic *CTNNA1* pathogenic variant carriers, who displayed DGC *foci* in the prophylactic gastrectomy and intramucosal *focus* on esophagogastroduodenoscopy specimens, with loss of catenin alpha-1 expression, suggesting that pathogenic variants in *CDH1* and *CTNNA1* may have similar implications in DGC risk [1,7]. The penetrance of pathogenic *CTNNA1* variants is still unclear, although Coudert and collaborators have recently published the first penetrance estimate for DGC in *CTNNA1* families as 49–57% at 80 years [10]. The association of *CTNNA1* pathogenic variants with increased LBC risk is also still inconclusive; although some studies have been associating *CTNNA1* and breast cancer [9,12,13,14], this association remains to be clarified since the subtype of these cancers was not specified. Therefore, breast cancer surveillance should be considered based on personal and family history [2].

The *CDH1* and *CTNNA1* genes are members of the cadherin and catenin family, respectively, encoding adherens junction proteins, and they are involved in intercellular cell adhesion [15]. The *CDH1* gene encodes the cadherin-1, a transmembrane protein, and its extracellular domain establishes interactions with cadherin-1 proteins on an adjacent cell, whereas its cytoplasmic domain binds to the members of the catenin protein family, such as catenin delta-1 and catenin alpha-1 via catenin beta-1, promoting protein stabilization [16]. The *CTNNA1* gene encodes the catenin alpha-1 protein, which forms a complex with catenin beta-1 to bind the cytoplasmic domain of cadherin-1 to the actin cytoskeleton by binding directly to the actin filaments [16,17,18]. This protein is essential for the function of the E-cadherin-catenin complex, and the inactivation of catenin proteins and disruption of cytoplasmic catenin-cadherin binding results in the inability of cadherin-1 to establish cell-to-cell adhesion [17]. The involvement of the catenin delta-1 protein in cell adhesion raises the question of its implication in GC predisposition. The catenin delta-1 protein is encoded by the *CTNND1* gene, interacts with the juxtamembrane domain of cadherin-1 and regulates the stability of cell–cell adhesion by controlling the retention of cadherin-1 at the cell surface, being crucial to the maintenance and stability of cell adhesion [16]. A single study has been reported that attempted to identify germline variants in the *CTNND1* gene and no pathogenic variants were found in the few families tested [19]. 

The International Gastric Cancer Linkage Consortium (IGCLC), in 1999, defined clinical criteria to select patients for the screening of *CDH1* germline variants [20]. Since then, these criteria have been updated several times [21,22] and, in 2020, the IGCLC published updated and less restrictive testing recommendations [2]. Moreover, according to these new guidelines, *CTNNA1* testing should be considered when no *CDH1* pathogenic variant has been identified [2]. Despite these updates, the guidelines remain unclear if mixed GC, which harbors the two histological components, intestinal and diffuse, should or should not be considered in the HDGC testing criteria [23,24]. In fact, the IGCLC guidelines are very clear about not considering the intestinal type for *CDH1*/*CTNNA1* testing, as this cancer type is not part of the syndrome [2], but they provide no guidance as regards to mixed GC.

We aimed to evaluate the contribution of *CTNNA1* and *CTNND1* germline variants to HDGC, as well as to compare the frequencies of *CDH1* and *CTNNA1* (and eventually *CTNND1*) germline variants in patients/families with DGC with those with mixed GC to evaluate the pertinence of genetic testing of these genes in the latter.

## 2. Materials and Methods

### 2.1. Patients and Samples

This study included 212 index cases; 193 patients who were referred to the Genetics Department of the Portuguese Oncology Institute of Porto (IPO Porto) between 2012 and 2021 and 19 patients from the Portuguese Oncology Institute of Lisbon referred to genetic counseling between 2014 and 2019. Of the 212 families, 145 met the IGCLC 2015 or 2020 clinical criteria for HDGC testing (5 of the index patients had LBC and all the others had DGC); among the 145 patients that met the IGCLC 2020 criteria, 26 patients did not fulfill the IGCLC 2015 criteria. Germline *CDH1* testing had been performed in these 145 index patients as part of standard clinical genetic testing. The remaining 67 patients were affected with mixed GC and would have met the IGCLC 2015 or 2020 testing criteria if this histological type had been considered for testing in the same way as DGC. 

This study was approved by the Ethics Committee of IPO Porto (reference number CES 351/018) and samples for genetic testing were obtained after genetic counseling and informed consent. 

Genomic DNA was extracted from peripheral blood samples according to standard protocols.

### 2.2. Next Generation Sequencing

*CDH1* (NM_004360.5) gene analysis was performed in 67 index cases with mixed GC by next-generation sequencing (NGS), using the TruSight Cancer panel (Illumina, Inc., San Diego, CA, USA), with library preparation performed according to the manufacturer’s protocol, and the sequencing reaction was carried out using a standard flow cell in the MiSeq platform (Illumina, Inc.) in 2 × 150 bp paired-end runs. Sequencing alignment and variant analysis were performed using a bioinformatics pipeline previously validated by our group [25]. In brief, alignment and variant calling were carried out using NextGENe (v2.4, Softgenetics, State College, PA, USA). NGS as described above had been used as part of clinical care for the 145 index cases tested for *CDH1*.

Sequencing of the *CTNNA1* (NM_001903.5) and the *CTNND1* (NM_001085458.1) genes was performed in 208 cases (with negative *CDH1* testing) using a customized QIASeq Targeted DNA Panel (Qiagen, Antwerp, Belgium). Library preparation was performed according to the manufacturer’s instructions and sequencing was carried out using a micro flow cell in the MiSeq Illumina sequencer (Illumina, Inc.) in a 2 × 150 bp paired-end run. Sequencing alignment and variant calling were performed using QIAGEN’s GeneGlobe data analysis portal (Qiagen). The .vcf files were imported to GeneticistAssistantTM software 1.8 (Softgenetics) for variant annotation. All variants with a variant allele frequency (VAF) less than 10%, minor allele frequency (MAF) greater than 1% and intronic variants at more than 12 bp away from exon–intron boundaries were excluded. For *CDH1* analysis, variants with a frequency higher than 5% in our in-house database were also excluded. For MAF filtering, data were obtained from the 1000 Genomes Project (1000 G; Based on Project Phase III Data), Genome Aggregation Database (gnomAD) and Exome Aggregation Consortium (ExAC) databases.

### 2.3. Immunohistochemistry

Catenin alpha-1 protein expression was evaluated by immunohistochemistry (IHC) in section from a representative formalin-fixed and paraffin-embedded tumor tissue block. Briefly, a mouse monoclonal antibody against catenin alpha-1 (clone EP1793Y, Abcam, Cambridge, UK) was incubated at 1/200 for 20 min, and staining was performed with the Leica Bond-III instrument according to the manufacturer’s instructions. Immunostained slides were evaluated by a pathologist. Diffuse gastric tumors known to be negative for *CTNNA1* and *CDH1* germline variants were used as controls to assess the specificity of the staining.

## 3. Results

**Figure 1:**
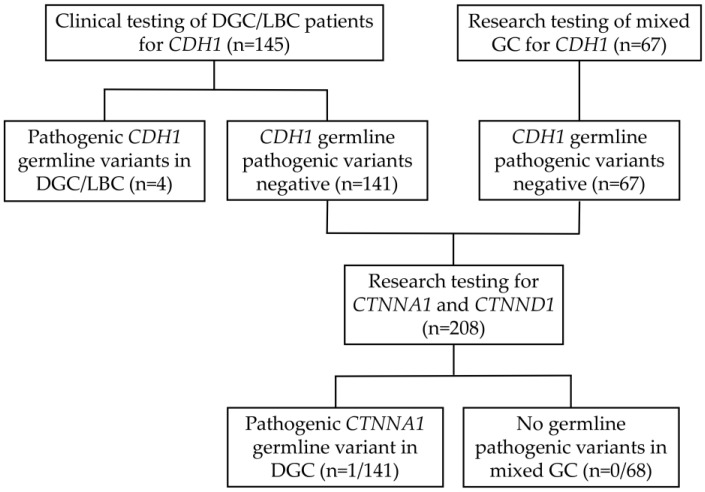
Flow chart representing patient inclusion and subsequent germline analysis for *CDH1*, *CTNNA1* and *CTNND1* genes.

**Figure 2:**
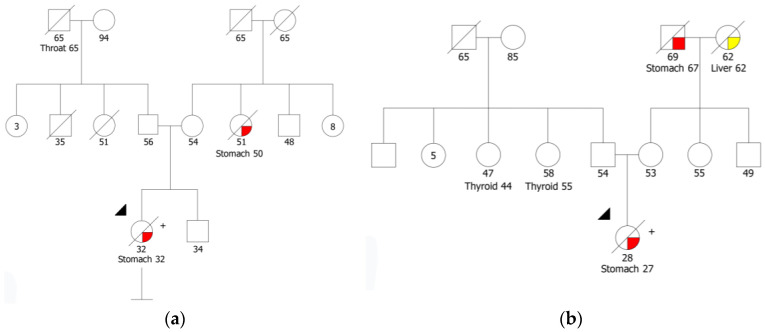
(**a**) Pedigree of the HDGC family with the *CDH1* variant c.377del p.(Pro126ArgfsTer89); (**b**) Pedigree of the index patient identified with the *CTNNA1* variant c.1426C>T p.(Gln476Ter).

A total of 212 patients were included in this study; 145 of these patients met IGCLC 2020 clinical criteria for HDGC testing and the remaining 67 patients were diagnosed with mixed GC. The *CDH1* gene had been analyzed in the 145 patients that fulfilled the IGCLC criteria as part of standard clinical genetic testing prior to this study and *CTNNA1* gene sequencing was performed in all patients who had tested negative for *CDH1* pathogenic germline variants (Figure 1). Four families have previously been identified with three pathogenic variants in the *CDH1* gene (Table 1). Three of these families were already reported [26,27], two harboring the recurrent splicing variant c.1901C>T, which was identified in two women diagnosed with LBC at 38 and 48 years old, and the third was the c.1565+1G>A variant identified in a man with a cleft lip and diagnosed with DGC at 63 years old. Lastly, the variant c.377del p.(Pro126ArgfsTer89) was identified in a woman diagnosed with DGC at 32 years old (Figure 2a). This variant is classified in ClinVar (ID 406616) as pathogenic and has already been reported in the literature [28].

**Figure 3:**
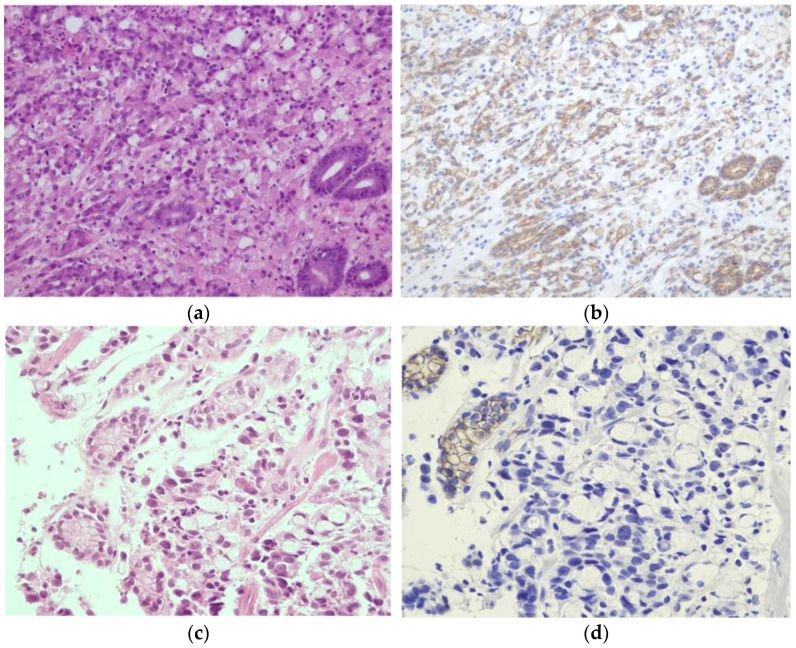
Microscopic findings showing hematoxylin and eosin (H&E) staining in the left column and immunohistochemistry for catenin alpha-1 in the right column. (**a**,**b**) Diffuse gastric cancer in a patient who does not carry *CTNNA1* germline variants. Catenin alpha-1 expression is retained in both normal and tumor cells (original magnification, ×200). (**c**,**d**) Gastric biopsies of the *CTNNA1* c.1426C>T p.(Gln476Ter) variant carrier show signet ring cells infiltrating the mucosa. Catenin alpha-1 was detectable in normal epithelial structures (e.g., the glands of the stomach), but the expression was completely lost in the tumor cells (original magnification, ×400).

The *CTNNA1* germline variant c.1426C>T was found in 1 of 141 (0.7%) patients meeting 2020 IGCLC criteria who had previously tested negative for *CDH1* deleterious variants. This is a nonsense variant predicted to lead to premature termination of translation and, consequently, to a truncated protein, p.(Gln476Ter), or to a nonsense-mediated mRNA decay. This variant was identified in a woman diagnosed with a poorly differentiated adenocarcinoma with signet ring cells at age 27 (Figure 2b). There are no *CTNNA1* variant classification guidelines; however, Lobo and collaborators classified all variants described in the literature following the *CDH1* ACMG/AMP guidelines [29,30]. Following these guidelines, the variant identified in our study is classified as pathogenic, using the criteria PVS1 (nonsense variant), PS4 supporting (one family meeting HDGC criteria) and PM2 (variant absent from population databases). We performed immunohistochemistry (IHC) to assess catenin alpha-1 protein expression in the biopsy sample of the *CTNNA1* pathogenic variant carrier. IHC revealed the loss of catenin alpha-1 expression in the gastric adenocarcinoma and preserved catenin alpha-1 expression in the normal epithelial structures (Figure 3d). We also performed IHC in gastric tumors of ten index cases from this series known to be wild-type for the *CDH1* and *CTNNA1* genes and for whom tumor specimens were available, and catenin alpha-1 expression was retained in both normal and tumor cells (Figure 3b). Regarding family history, the proband’s parents are not affected; the maternal grandfather had stomach cancer at age 67 and the maternal grandmother had liver cancer at 62, both deceased. Two paternal cousins were diagnosed with thyroid cancer at 44 and 55 years old, respectively. To date, it was not possible to perform segregation analysis or test the parents of the proband. 

None of the 67 cases with mixed GC carried pathogenic variants in either the *CDH1* or the *CTNNA1* genes. Furthermore, no deleterious *CTNND1* variant was detected in the 208 patients without the *CDH1* pathogenic germline variants. We identified four missense and two synonymous variants in the *CTNND1* gene classified as variants of uncertain significance (VUS) following the ACMG guidelines [29], both in patients with diffuse and mixed gastric cancer subtypes (Table 2). 

## 4. Discussion

Until recently, *CDH1* was the only known susceptibility gene associated with HDGC, but, in 2020, the IGCLC recognized *CTNNA1* as an HDGC predisposition gene and recommended *CTNNA1* testing when no *CDH1* pathogenic variant has been identified [2]. On the other hand, the contribution of *CTNND1* germline variants for GC predisposition is unknown. Furthermore, the IGCLC still does not clarify if mixed GC with a diffuse component should or should not be considered in the clinical criteria for genetic testing.

Prior to this study and as part of clinical care, the 145 index cases that fulfilled the HDGC testing criteria had been analyzed for *CDH1* pathogenic variants, and four families were identified with three pathogenic variants in the *CDH1* gene (Table 1). The prevalence of pathogenic *CDH1* variants in our patients with HDGC genetic testing criteria is approximately 3% (4/145). The prevalence of *CDH1* pathogenic variants in suspected HDGC families varies widely in the literature, which can be mainly explained by the different GC incidences between countries [4]. High-incidence countries, such as Portugal, display a lower frequency of molecularly confirmed HDGC [4,31,32], which can explain the relatively low prevalence of *CDH1* deleterious variants we present here. In this study, we analyzed the two candidate genes, *CTNNA1* and *CTNND1*, in the remaining 141 index cases that were negative for *CDH1* pathogenic variants. We identified a novel nonsense variant in *CTNNA1*, c.1426C>T p.(Gln476Ter) in a woman diagnosed with a poorly differentiated adenocarcinoma with signet ring cells at age 27 (Figure 2b). We used the *CDH1* ACMG/AMP guidelines to classify this variant as pathogenic, as described by Lobo et al. [29,30]. The IHC showed loss of catenin alpha-1 expression in the tumor cells and preserved catenin alpha-1 expression in the normal gastric epithelium of the *CTNNA1* variant carrier. Furthermore, catenin alpha-1 expression was retained in the tumors of ten proband cases, known to be wild-type for *CTNNA1* and *CDH1* genes, suggesting that catenin alpha-1 expression loss in DGC can be predictive of a *CTNNA1* pathogenic variant. Loss or decrease in catenin alpha-1 expression in tumors or biopsy samples of *CTNNA1* carriers has also been demonstrated by several authors [3,7,9]. These observations suggest that a second hit inactivating mechanism occurred somatically at the *CTNNA1* gene; however, the tumor sample was not available to search for this mechanism. Although the prevalence of *CTNNA1* pathogenic variants is not well-established, Lobo et al. suggested that pathogenic variants in *CTNNA1* seem to account for less than 2% of the families fulfilling the HDGC testing criteria [11]. The prevalence of pathogenic *CTNNA1* variants in our families fulfilling 2020 HDGC testing criteria is approximately 1%, which is similar to the frequency described in the literature [11].

According to the latest HDGC guidelines, *CTNNA1* is considered to be a predisposition gene, along with *CDH1*. Annual endoscopy screening following the Cambridge protocol is recommended in asymptomatic carriers and total gastrectomy should be considered depending on the results of the biopsies and the penetrance of DGC in the family [2]. The risk of GC in carriers of pathogenic *CTNNA1* variants is not well defined, but a recent study evaluated DGC risk in *CTNNA1* carriers and estimated a cumulative risk of DGC at 80 years of 49–57% [10]. The association of *CTNNA1* pathogenic variants with high risk of LBC is also still uncertain; therefore, breast cancer surveillance should be considered based on personal and family history [2]. 

Beyond HDGC predisposition, the *CTNNA1* and *CDH1* genes have been associated with macular dystrophy patterned [33,34] and cleft lip/palate and blepharocheilodontic syndrome (BCD) [35,36], respectively. The *CTNND1* gene has also been associated with BCD [35,36,37], and the involvement of another cadherin–catenin complex gene in cleft palate and BCD, in addition to its important function in the maintenance and stability of cell adhesion, which makes this gene an ideal candidate for GC predisposition [38]. We therefore analyzed the *CTNND1* gene in 208 patients included in this study, but no pathogenic variants were found in the 141 patients with the 2020 IGCLC criteria negative for *CDH1* variants. Our negative findings support those of Schuetz et al. [19] in 22 families. However, we identified six VUS in the *CTNND1* gene, both in patients with diffuse and mixed gastric cancer subtypes. Here, we report the largest series to date that tested for *CTNND1* variants in suspected HDGC patients, but even larger studies will be needed to clarify if the *CTNND1* gene has any role in GC predisposition. 

A proportion of gastric carcinomas display a mixed phenotype presenting the two histological components, intestinal and diffuse [23,24]. Interestingly, while intestinal-type GC has been completely excluded from the HDGC spectrum [20,21] and the IGCLC guidelines are very clear to not test the patients with this histological type, these guidelines do not indicate whether the mixed type should be considered in the criteria for *CDH1* germline variant testing. In this study, we analyzed the *CDH1*, *CTNNA1* and *CTNND1* genes in 67 cases affected with mixed GC and did not find any pathogenic *CDH1, CTNNA1* or *CTNND1* variant. To our knowledge, there are no pathogenic *CDH1* variants described in cases with mixed GC in the literature [39,40]. Furthermore, the findings of Machado et al. are in favor of a clonal origin of mixed GC and suggest that an isolated-cell histological component may be originated due to the occurrence of *CDH1* somatic variants [41]. Therefore, there is currently no evidence that supports including this tumor type in germline testing of the *CDH1* or *CTNNA1* genes. However, studies in larger series of mixed GC are necessary to clarify this issue. 

## 5. Conclusions

In this study, a deleterious *CTNNA1* germline variant was found in 0.7% (1/141) of patients meeting the 2020 IGCLC criteria, as compared to the rate of 2.8% of *CDH1* deleterious variants. Despite the relatively low frequency of HDGC families associated with pathogenic germline variants in *CTNNA1*, screening for this gene should be conducted for *CDH1*-negative HDGC families, as recommended in the latest IGCLC guidelines [2]. This will enable the identification of additional *CTNNA1* families, allowing more accurate penetrance estimates and a better-defined disease spectrum. The role of *CTNND1* variants in inherited gastric cancer predisposition is still unclear.

## Figures and Tables

**Table 1:** Germline pathogenic variants in the *CDH1* and *CTNNA1* genes identified in families fulfilling genetic testing criteria.

Family	Gene	HGVS Coding	HGVS Protein	Gastric Cancer Histology	Breast Cancer Type	Age of Diagnosis	HDGC Criteria
1 *	*CDH1*	c.1901C>T	p.(Ala634Val)	-	Lobular breast cancer	39	Yes
2 *	*CDH1*	c.1901C>T	p.(Ala634Val)	-	Lobular breast cancer	48	Yes
3 *	*CDH1*	c.1565+1G>A		Diffuse gastric cancer	-	63	Yes
4	*CDH1*	c.377del	p.(Pro126ArgfsTer89)	Diffuse gastric cancer	-	32	Yes
5	*CTNNA1*	c.1426C>T	p.(Gln476Ter)	Diffuse gastric cancer	-	27	Yes

* These families have previously been reported in other studies [26,27].

**Table 2:** Variants of uncertain significance identified in the *CTNND1* gene.

HGVS Coding	HGVS Protein	Variant Type	GnomAD Frequency	ClinVar Classification	ClinVar ID	ACMG Classification	Number of Samples	Gastric Cancer Histology
c.148G>C	p.Asp50His	missense	N/A	VUS	2391342	VUS	1	Mixed
c.202C>T	p.Arg68Trp	missense	0.0003848	VUS	1311724	VUS	1	Mixed
c.790C>T	p.Arg264Trp	missense	0.0000121	N/A	N/A	VUS	1	Mixed
c.1671C>A	p.Ala557=	synonymous	0.0000040	N/A	N/A	VUS	1	Diffuse
c.2130G>A	p.Lys710=	synonymous	N/A	N/A	N/A	VUS	1	Mixed
c.2474T>C	p.Val825Ala	missense	0.0000392	N/A	N/A	VUS	1	Diffuse

VUS, variant of uncertain significance; N/A, not available.

## Data Availability

The data presented in this study are available on request.
